# Supplementary material for: Evolutionary dynamics in the dispersal of sign languages
Source: R Soc Open Sci. 2020 Jan 22;7(1):191100. doi: 10.1098/rsos.191100 (PMC7029929; doi:10.1098/rsos.191100)
Supplement: Appendix I [file rsos191100supp1.pdf]

Supplementary Information for the Paper "Evolutionary Dynamics in the Dispersal of Sign Languages"

Justin M. Power, Guido W. Grimm, and Johann-Mattis List

December 2019

Contents

1 Organisation of the supplementary material **2**

2 Language selection and historical connections amongst sign languages **2**
2.1 Extant manual alphabets . . . 2
2.2 Historical manual alphabets . . . 3
2.3 Previously-reported historical connections amongst SLs . . . 4

3 Data preparation and curation **5**
3.1 Technical aspects . . . 5
3.2 Coding . . . 6

4 Phylogenetic analysis **7**
4.1 Reasons for exploratory data analysis and effects of incompatible signal . . . 7
4.2 Character mapping . . . 8
4.2.1 Explanation of annotations and abbreviations used for lingotypes/handshapes in character maps . . . 9
4.2.2 Comprehensive character map for sign languages up to 1840 . . . 10
4.2.3 Character map for sign languages from early 19th to mid-/late 20th century . . . 11
4.2.4 Character map for contemporary sign languages . . . 12
4.3 Split support . . . 13
4.4 Evolution of early manual alphabet forms . . . 17

1 Organisation of the supplementary material

The data was annotated with the help of the EDICTOR (List 2017) using a server-based version to ease collaboration. A link to the database can be found at `http://edictor.digling.org/?file=signalphabets&remote_dbase=signalphabets`. Since the database annotation process was in flux for some time, and may change in the future, we curate the data on GitHub, where it can be explicitly versionized (`https://github.com/lexibank/powerma`), and versions considered stable can be archived with Zenodo (`https://zenodo.org/record/3564465`, Version v1.0.2). The methods described in the study themselves along with the results and the code needed to convert the data into the formats required by the software packages are again curated on GitHub (`https://github.com/lingpy/sign-language-evolution-paper`), and have been archived with Zenodo (`https://zenodo.org/record/3564484`, Version v1.0.1).

2 Language selection and historical connections amongst sign languages

2.1 Extant manual alphabets

The table below shows the extant manual alphabets (MAs) of sign languages (SLs) and sources in our sample, with IDs taken from Figures 1 and 5 in the main text.

ID	Manual Alphabet	Abbrev	Glottolog	Source
23	Afghan Sign Language	ZEA	afgh1239	Afghan NAD 2001
24	Albanian Sign Language	AlbSL	alba1271	Albanian NAD 2019
27	American Sign Language	ASL	amer1248	Tennant et al. 1998, Lydell 2018
18	Australian Sign Language	Auslan	aust1271	Johnston 2014
30	Austrian Sign Language	ÖGS	aust1252	Lydell 2018
31	Brazilian Sign Language	LSB	braz1236	Lydell 2018
4	British Sign Language	BSL	brit1235	Brien 1992, Lydell 2018
32	Bulgarian Sign Language	БЖЕ(BZhE)	bulg1240	Lydell 2018
33	Catalan Sign Language	LSC	cata1287	Perelló et al. 1998
34	Croatian Sign Language	HZJ	croa1242	Šarac Kuhn et al. 2006, Lydell 2018
35	Czech Sign Language	CzSL	czec1253	Hudáková 2008
40	Danish Sign Language	DTS	dani1246	Fischer et al. 2019
70	Dutch Sign Language	NGT	dutc1253	Zwitserlood 2010
42	Estonian Sign Language	EVK	esto1238	Eesti Keele Instituut 2014
43	Finnish Sign Language	FinSL	finn1310	Kuurojen Liitto 1998
44	Flemish Sign Language	VGT	vlaa1235	Vlaams Gebarentaal Centrum 2012
48	French Sign Language	LSF	fren1243	Lydell 2018
49	French-Belgian Sign Language	LSFB	lang1248	Sonnemans 2016
52	German Sign Language	DGS	germ1281	Lydell 2018
53	Greek Sign Language	ΕΝΓ(ENG)	gree1271	Hatzopoulou 2008, Lydell 2018
54	Icelandic Sign Language	ÍTM	icel1236	Lydell 2018
22	Indian Sign Language	IPSL	indi1237	Lydell 2018
55	International Sign	IS	inte1259	Rubino et al. 1975, Lydell 2018
56	Irish Sign Language	ISL	iris1235	LeMaster 2002

57	Italian Sign Language	LIS	ital1275	Magarotto 1996, Lydell 2018
58	Jordanian Sign Language	LIU	jord1238	Hendriks 2008
59	Latvian Sign Language	LSL	latv1245	Latvian AD 2016, Lydell 2018
60	Lithuanian Sign Language	LGK	lith1236	Lietuvos 2012, Lydell 2018
61	Mexican Sign Language	LSM	mexi1237	Lydell 2018
20	New Zealand Sign Language	NZSL	newz1236	McKee et al. 2011
64	Norwegian Sign Language	NTS	norw1261	Statped 2016
65	Pakistan Sign Language	PSL	paki1242	Sabir Khan et al. 2014, Lydell 2018
66	Polish Sign Language	PJM	poli1259	Łacheta et al. 2016, Lydell 2018
16	Portuguese Sign Language	LGP	port1277	Lydell 2018
67	Quebec Sign Language	LSQ	queb1245	Fondation des Sourds du Québec 2019
68	Russian Sign Language	РЖЯ(RZhIe)	russ1255	Lydell 2018
73	Spanish Sign Language	LSE	span1263	Blanco 2009
74	Swedish Sign Language	STS	swed1236	Avdelningen för teckenspråk 2014
75	Turkish Sign Language	TİD	turk1288	Zeshan 2003
76	Ukrainian Sign Language	УЖМ(UZhM)	ukra1235	Lydell 2018

Table S1: Contemporary manual alphabets in our sample. NAD = National Association of the Deaf; AD = Association of the Deaf.

2.2 Historical manual alphabets

The following table shows the historical MAs and sources in our sample, with IDs taken from Figures 1 and 5 in the main text, and abbreviations taken from Table S1 in the Supplementary Information.

ID	Abbrev	Year	Location	Source
14	ASL	1821	New York, USA	Akerly 1821
25	ASL	1886	Washington D.C., USA	Gordon 1886
26	ASL	1918	Iowa, USA	Long 1918
12	DTS	1808	Copenhagen, Denmark	Castberg 1818
36	DTS	1871	Copenhagen, Denmark	Nyegaard 1871
37	DTS	1907	Copenhagen, Denmark	Jorgensen 1907
38	DTS	1926	Copenhagen, Denmark	Døvstumme-Radet 1926
39	DTS	1967	Copenhagen, Denmark	Plum et al. 1976
6	DGS	1821	Gmünd, Germany	Alle 1821
50	DGS	1909	Leipzig, Germany	Reuschert 1909
51	DGS	1916	Berlin, Germany	Riemann 1916
41	EVK	1988	Tallinn, Estonia	Toom 1988
19	ÍTM	1857	Akureyri, Iceland	Sigurðsson 1857
3	LSF	1799–1800	Paris, France	Anonymous 1800
45	LSF	1803	Paris, France	Sicard 1803
46	LSF	1815	Paris, France?	De Ladebat 1815
47	LSF	1856	Paris, France	Pelissier 1856
10	LSE	1815	Madrid, Spain	Martí 1815
71	LSE	1845	Madrid, Spain	Ballesteros & Villabrille 1845

72	LSE	1859	Madrid, Spain	Carderera 1859
21	LSB	1875	Rio de Janeiro, Brazil	Da Gama 1875
7	LIS	1897	Milan, Italy	Fornari 1897
9	MJ	1827	Vác, Hungary	Schwarzer 1827
8	NGT	1790	Groningen, Netherlands	Mörser & Guyot 1790
69	NGT	1820	Groningen, Netherlands	Van Heijningen Bosch 1820
17	NTS	1893	Oslo, Norway	Svendsen 1893
62	NTS	ca. 1900	Trondheim, Norway	Bruun 1900
63	NTS	1955	Trondheim, Norway?	Norske Døves Landsforbund 1955
5	ÖGS	1786	Vienna, Austria	May 1789
28	ÖGS	1823	Vienna, Austria	Venus 1823
29	ÖGS	1839	Vienna, Austria	Czech 1839
15	PJM	1879	Warsaw, Poland	Hollaka & Jagodzińskiego 1879
13	STS	1866	Stockholm, Sweden	Paulsson 1866
11	РЖЯ	1835	St. Petersburg, Russia	Fleri 1835
1	Yebra	1593	Madrid, Spain	De Yebra 1593
2	Bonet	1620	Madrid, Spain	Bonet 1620

Table S2: Historical manual alphabets in our sample.

2.3 Previously-reported historical connections amongst SLs

The following table provides an overview of previously-reported historical connections amongst SLs in the study's sample. In reporting historical connections amongst SLs, sources may not have intended to make explicit claims about language classifications. Years reflect the first incidence of contact when known.

Language	Connection	Year	Source
Afghan SL	America, Jordan	1992–95	Lee 1999
Albanian SL	Russia	1963	Hoyer 2007
American SL	France	1817	Tabak 2006
Australian SL	England, Scotland	1825	Schembri et al. 2010
	Ireland	1875	Schembri et al. 2010
Austrian SL	France	1777	Schalber 2015
British SL	France	1760	Eriksson 1998
Brazilian SL	France	1856	Xavier et al. 2015
Bulgarian SL	unknown		
Catalan SL	Italy	1800	Von der Lieth 1967
	Spain	not reported	Parkhurst et al. 2003
Croatian SL	Austria	not reported	Šarac Kuhn et al. 2006
Czech SL	Austria	1786	Filippová et al. 2016
Danish SL	Austria, Germany, France	1802–05	Bergman et al. 2010
Dutch SL	France	1790	Erikkson 1998
Estonian SL	Sweden, Finland, Russia	not reported	Eberhard et al. 2019, Bickford 2005
Finnish SL	Sweden	1846	Bergman et al. 2010
Flemish SL	France	1820	De Clerck 2009
French SL	Spain	ca.1759–84	Plann 1997
French-Belgian SL	France	1819	Kusters 2017
German SL	Independent	1778	Boyes Braem et al. 2010

Greek SL	America	1923	Lampropoulou 1994
	France	not reported	Sapountzaki 2015
Hungarian SL	Austria	1802	McCagg 1993
Icelandic SL	Denmark	1867	Thorvaldsdóttir et al. 201
Indian SL	England	1885	Vasishta 2011
Irish SL	England	1816	Leeson et al. 2015
	France	1846	Leeson et al. 2015
Italian SL	France	1784	Geraci 2015
	Austria	not reported	Corazza et al. 2008
Jordanian SL	Lebanon	not reported	Hendriks et al. 2009
Latvian SL	unknown		
Lithuanian SL	unknown		
Mexican SL	France, Brazil	1867	Quinto-Pozos 2008
New Zealand SL	England	1868	Schembri et al. 2010
	Ireland	1944	Schembri et al. 2010
Norwegian SL	Denmark	1825	Greftegreff et al. 2015
Pakistan SL	unknown		
Polish SL	Austria, Germany, Russia	not reported	Wojda 2010
	France	1817	Van Cleve 1987
Portuguese SL	Sweden	1823	Bergman et al. 2010
Quebec SL	America	1831	Parisot et al. 2015
	France	ca.1850	Parisot et al. 2015
Russian SL	Austria, France	1806–10	Abramov 1993
Spanish SL	France	1805	Von der Lieth 1967
Swedish SL	Independent	1809	Bergman et al. 2010
Turkish SL	England	1953	Zeshan 2003
Ukrainian SL	Russia	not reported	Bickford 2005
	Austria	1805	Viktorivna 2010

Table S3: Previously-reported historical connections amongst SLs.

3 Data preparation and curation

3.1 Technical aspects

To edit the data in a machine- and human-readable way, we used the EDICTOR application (List 2017), which was originally designed for spoken languages and phonetic transcriptions. To annotate morphologically similar handshapes, we used the "full cognate" annotation schema, which assumes that cognacy is a transitive relation applying to a full form in a binary fashion (two forms are either cognate or not). To export the data to the NEXUS format, we made use of LingPy (List et al. 2018). The accompanying repository with data-dump and source code shows how LingPy can be used for data conversion.

3.2 Coding

ID	DOCULECT TRANSLATION	CONCEPT	HANDSHAPE 1	COGID	NARROW CONCEPT	YEAR
1604	American Sign Language	h	[illegible]	145	h	1821
64	American Sign Language	h	[illegible]	145	h	
1527	Austrian Sign Language	h	[illegible]	118	h	1823
200	Austrian Sign Language	h	[illegible]	145	h	
143	Estonian Sign Language	h	[illegible]	113	h	
149	Estonian Sign Language	h	[illegible]	299	n	
1449	German Sign Language	h	[illegible]	118	h	1820
229	German Sign Language	h	[illegible]	145	h	
283	Latvian Sign Language	h	[illegible]	299	h	
1235	Russian Sign Language	h	[illegible]	299	н	1835
402	Russian Sign Language	h	[illegible]	299	н	
1610	American Sign Language	n	[illegible]	255	n	1821
70	American Sign Language	n	[illegible]	137	n	
1532	Austrian Sign Language	n	[illegible]	255	n	1823
206	Austrian Sign Language	n	[illegible]	137	n	
153	Estonian Sign Language	n	[illegible]	255	p	
1454	German Sign Language	n	[illegible]	255	n	1820
235	German Sign Language	n	[illegible]	137	n	
292	Latvian Sign Language	n	[illegible]	255	n	
1237	Russian Sign Language	n	[illegible]	255	п	1835
404	Russian Sign Language	n	[illegible]	137	п	

Taxon	145	118	113	299	255	137
American 1821	1	0	0	0	1	0
American SL	1	0	0	0	0	1
Austrian 1823	0	1	0	0	1	0
Austrian SL	1	0	0	0	0	1
Estonian SL	0	0	1	1	1	0
German 1820	0	1	0	0	1	0
German SL	1	0	0	0	0	1
Latvian SL	0	0	0	1	1	0
Russian 1835	0	0	0	1	1	0
Russian SL	0	0	0	1	0	1

Figure S1: Simplified coding example for handshapes representing Latin <*h*>, <*n*>, and <*p*>, and Cyrillic <н> and <п>.

Here, we provide a second example of our coding methods in cross-alphabet comparisons. Coding is more complex when there is a mismatch across alphabet types of graphemic forms and sounds represented. Figure S1 exemplifies our coding for ten MAs of handshapes representing Latin <*h*>, <*n*>, and <*p*>, as well as Cyrillic <н> and <п>. Consider, first, the overlap in sounds represented across the two alphabets. Latin <*h*> represents the voiceless glottal fricative (IPA [h]), a sound for which Cyrillic has no corresponding letter; Latin <*n*> and Cyrillic <н> represent the voiced alveolar nasal (IPA [n]); and Latin <*p*> and Cyrillic <п> represent the voiceless bilabial stop (IPA [p]). However, when considering the forms of the graphemes, the two alphabets overlap in different ways: there are similarities between the forms of Latin <*h*> and Cyrillic <н>, as well as Latin <*n*> and Cyrillic <п>.

The left side of the figure shows our coding of handshapes representing the letters described above. We coded the handshapes representing Latin <*h*> in contemporary Austrian SL, German SL, and American SL, as well as historical American SL (American 1821), as similar and assigned them character ID 145 (in the column "CogID"). Handshapes for Latin <*h*> in historical Austrian SL (Austrian 1823) and German SL (German 1820) were coded as similar and assigned ID 118. We chose to compare handshapes for Cyrillic <н> in historical (Russian 1835) and contemporary Russian SL with the handshapes representing Latin <h> mentioned above. Due to historical connections between Austrian-trained educators and the establishment of deaf education in Russia in the early 19th century (Abramov 1993, Williams and Fyodorova 1993), we reasoned that the similarity in handshape and graphemic forms for Latin <*h*> and Cyrillic <н> in Austrian 1823 and Russian 1835 are due to the adaptation of the Austrian h-handshape in the Russian SL MA to represent Cyrillic <н>. The alternative comparison, in which handshapes for Cyrillic <н> are compared with non-homologous handshapes representing Latin <*n*>, is a possibility but is, in our view, an incorrect approach based on the historical record. Therefore, we coded the historical and contemporary Russian handshapes in the <*h*>-comparison, which can be seen by observing the "Concept" and "Narrow concept" columns. The handshapes representing Cyrillic <н> in Russian 1835 and contemporary Russian SL were coded as similar and assigned ID 299. Similarly, we compared the handshape forms representing Latin <*n*> in Estonian SL and <*h*> in Latvian SL to the forms in the <*h*>-comparison. That the handshapes in Estonian SL and Latvian SL are homologous to the Russian SL forms for Cyrillic <н> is likely due to the history of deaf education in the former Soviet Union and the use of Russian SL in Estonia. In addition, as we have described, the graphemic form of Latin <*h*> is similar to the form of Cyrillic <н>, and both Latin <*n*> and Cyrillic <н> represent the alveolar nasal (IPA [n]). We coded the forms for <*n*> in Estonian SL and <*h*> in Latvian SL as similar to the Russian SL form and assigned them the character ID 299.

Next, historical examples of American SL (American 1821), Austrian SL (Austrian 1823), and German SL (German 1820), as well as contemporary Latvian SL, use similar handshapes for Latin <*n*> and were

assigned ID 255. Handshapes for Latin <*n*> in contemporary American SL, Austrian SL, and German SL were coded as similar and assigned ID 137. (Note that the difference in HamNoSys symbols used to code handshapes for Latin <*n*> in American and German SLs, on the one hand, and Austrian SL, on the other, reflect only slight differences in the position of the thumb.) The cases of contemporary and historical Russian SL, as well as Estonian SL, were more complex. Using similar reasoning to that described above for comparing handshapes for Latin <*h*> and Cyrillic <н>, we compared the Austrian 1823 handshape representing Latin <*n*> with the Russian 1835 handshape for Cyrillic <п>, the latter grapheme representing the voiceless bilabial stop (IPA [p]). We included the Russian 1835 handshape for <п> in the <*n*>-comparison and coded the handshape with ID 255. The contemporary Russian SL handshape for <п> differs slightly from the Russian 1835 example, with greater bending of the index and third fingers, and was assigned ID 137. Estonian SL reversed the process just described, taking the Russian SL handshape for Cyrillic <п> to represent Latin <*p*>. Thus, we included the Estonian SL handshape for <*p*> in the <*n*>-comparison, assigning ID 255. In addition, because the Estonian SL handshape for <*n*> was coded in the <*h*>-comparison, and because Estonian SL also has a handshape representing Latin <*h*>, Estonian forms were coded twice in the set of character IDs for the <*h*>-comparisons. Notice that there are at least two reasonable options for organising the comparison with respect to Estonian SL: (i) include the Estonian SL handshape representing <*n*> in the <*h*>-comparison along with the Russian SL handshape representing <н>, in order not to lose the homologous relationship; or (ii) include the Estonian SL handshape in the <*n*>-comparison and the Russian SL handshape in the <*h*>-comparison. We decided to take the first approach because the homologous relationship between the two handshapes seems clear, and the second coding approach would exaggerate the distance between the two languages. The Estonian SL handshape for Latin <*h*> is unlike any other form and was assigned ID 113.

4 Phylogenetic analysis

4.1 Reasons for exploratory data analysis and effects of incompatible signal

We assume that, in the case of language, evolutionary history does not strictly follow a tree model, but includes reticulation as a result of borrowing and, in the case of MAs, partial or complete replacement by standardisation or due to complex socio-political constraints. In addition, we expect a substantial amount of positive selection due to articulatory and perceptual pressures within the framework of a limited number of possible morphologies (handshapes), potentially resulting in homoplasy, as similar handshapes evolved or were conceived independently from each other. Homoplasy will provide support for topological alternatives, outcompeting those best reflecting the evolutionary history of SLs. Finally, our data set includes historical and contemporary MAs (i.e., potential ancestors and their descendants), a situation poorly handled by trees and best by neighbour-nets (NNets) (Spencer et al. 2004). Each aspect can add an additional evolutionary/historical dimension, and a tree is, per se, a 1-dimensional graph.

For instance, if A, B, and C have a common origin only expressed by similarity of B to both A and C, it's impossible to find a correct tree. The NNet can handle incompatible signal to some degree (B will be placed between A and C) but is restricted to distance matrices, which can be strongly biased by missing data artefacts (here, the number of CogIDs applicable only for a subset of the SLs); and they are planar, 2-dimensional graphs. If a taxon or lineage shares traits with more than two distinct, unrelated lineages (e.g., if B borrowed from the not directly related D), one of these relationships will get lost in the graph. The limitation to two dimensions is the reason for the differences between the all-inclusive NNet in the main text (Figure 3) and the time-filtered NNets in the main text (Figure 4), both based on the same distance matrix (e.g., regarding the placement of Russian 1835). Consensus networks (CNets) are *n*-dimensional, that is, if the data reflect *n* different topologies, they will all be represented in the CNet. For the A-B-C-D example, we may find support for three partly incompatible splits: A + B, B + C, B + D. If the common origin of A, B, and C is well reflected in the character-matrix, we will get high support for a fourth split,

A + B + C, which competes with B + D, and both will be seen in the CNet. The NNet will, in contrast, place B between A + C and D (aspect-wise correct), and the tree will place either (i) B as sister to D and both as sister to A + C, or (ii) D as sister to A + B + C, which is equally incorrect. However, CNets are based on a tree sample, hence, all limitations that apply for using tree-inference to reconstruct the history of (here) SLs, apply also for each inferred pseudoreplicate tree during bootstrapping (BS) (here, 10,000 BS pseudoreplicates).

In addition to general branching artefacts, we will have more or less random branching patterns (neighbour-joining- and maximum likelihood-BS (NJ-BS and ML-BS) replicates are always fully-resolved trees, no branch has zero length, no polytomies). The use of different optimality criteria allows for testing the stability of potential relationships seen in the BS tree samples on which the BS-support CNets are based under different assumptions: NJ-BS CNets show the robustness of signal based on overall similarity (or dissimilarity), minimising the effect of single, potentially misleading characters, but also inflicting relationships based on dissimilarity to everything else (including long-branching artifacts, LBA); MP-BS CNets, also affected by LBA, provide the most-conservative, but often also least-discriminating result under the assumption that all character changes (mutations) have exactly the same probability, an assumption that must be wrong for our data; and ML-BS CNets optimise a model that allows for between-character variation but which runs the risk of over-weighting certain character splits. If the CNets converge and support a neighbourhood seen in the NNet (see Section 4.3, Fig. S5), it can be considered a data-unbiased result; if they deviate from or contrast with each other (compare Fig. S6A–D in Section 4.3), the resulting competing alternatives may be biased by unrepresentative pairwise distance profiles due to missing data, or by inferred character mutations that strongly depend on the assumed model. Given the complexity of character similarity and historical pathways of SLs, it is impossible to judge, in such cases, which optimality criterion gives a better reflection of the true situation. However, it is safe to assume that the true situation is one of the preferred alternatives, if it can be explained by a single tree; or that the different preferred alternatives show different aspects of the true situation, if too complex for a single tree.

4.2 Character mapping

The figures in this section show character maps based on the binary sequences encoding for the basic concepts shared among all Latin alphabets, i.e., the standard set of 26 letters from <*a*> to <*z*>.

The applied mapping procedure is trivial; in principle it follows Ockham's Razor, parsimony, and the logic behind parsimony-based haplotype networks, specifically Median networks (Bandelt et al. 2000). First, we consider each unique binary sequence encoding for each MA form of a concept as a "lingotype", in analogy to haplotypes in biology. Each lingotype is the binary representation of a handshape. Second, we infer hypothetical mutational pathways by devising simple, (full) median networks for the lingotypes of each concept. By definition, a full median network includes all parsimonious solutions to evolve haplotypes (here: binary sequences = lingotypes) into each other. In the case of our data, a concept encoded by two binaries (each one reflecting a CogID) will usually have two lingotypes (differentiated MA forms): a lingotype A with "1 0" and a lingotype B with "0 1", which differ by a single 2-step mutation (replacement of one CogID by the other). The resulting median network is a two-taxon unrooted tree (i.e., a 1-dimensional graph) with a single branch. A concept encoded by three binaries resulting in three unique, mutually exclusive lingotypes (A = "1 0 0", B = "0 1 0", C = "0 0 1") results in a triangle, a 2-dimensional graph, in which each lingotype differs from any other by a single replacement of one binary (CogID) by another. Similarly, a four binary-long concept with four mutually exclusive lingotypes would result in a tetrahedron, a 3-dimensional structure, and so on. In some cases, lingotypes combine two CogIDs. Such lingotypes are treated as medians in the context of median networks. For example, a concept encoded by two binaries but with three lingotypes in the taxon set (A = "1 0", B = "0 1", X = "1 1") would result in a simple linear tree: A $\leftrightarrow$ X $\leftrightarrow$ B ($\leftrightarrow$ representing gain or loss of first and second CogID).

In the last step, the resulting mutation patterns are mapped visually, by eye and hand, on the time-taxon-filtered NNets, again using the logic behind parsimony-based haplotype networks to identify potential back-

mutations (e.g., borrowing) within a lineage. For the triangular example above (3 binary-long lingotypes A, B, C), if OTUs (taxonomic units, "leaves") with lingotype C form a subgroup deeply embedded in the lingotype A neighbourhood, the deduced mutational network mapped on the graph would not be the original triangle but a tree: B ↔ A ↔ C (in biological terms: lingotype C would be treated as a satellite haplotype of lingotype A). The map would include the edge-related annotations: "→ A", "→ B", "A → C". If lingotype A were widespread and lingotypes B and C restricted to terminal neighbourhoods in the NNet, the deduced mutational network would be the same: B ↔ A ↔ C, with the difference that A in this scenario is the ancestor of both B and C (a "median" in the concept of median and other parsimony networks). Thus, the map would indicate that A is ancestral, and the edges relating to the lingotype B and C neighbourhoods would be annotated as "A → B" and "A → C". When deduced, we consider not only the lingotypes and resulting character cliques mapped on the networks but also the age and country of the MA.

For instance, for the concept <*k*> in the oldest MAs set (Figure S2) we have three mutually exclusive binary sequences (lingotypes, addressed and abbreviated as "Dutch" = "Du.", after the earliest MA showing this handshape, Dutch 1790, vs. "†Fr." vs. "At."; full list of lingotype labels is provided in Table S4 below). The full median network for handshapes of the concept <*k*> is a triangle: to change from one lingotype to another requires two steps (loss of the CogID defining the one handshape and gain of the CogID defining the other handshape). <*k*> is not defined for the Spanish-origin group including the oldest MAs (Bonet, Yebra) that influenced the first MAs in all groups. Hence, the Dutch version can be inferred as one primitive, commonly-shared handshape that evolved from an unknown source ("→Du."), possibly competing with the extinct French handshape "→ †Fr." in the nearly as old French 1800 and 1803 MAs. The original Austrian-origin group handshape (lingotype "At.") is inferred to have replaced the Dutch version ("Du. → At."): the neighbourhood linked to the Austrian handshape is embedded in a much larger neighbourhood including earliest Danish and Russian MAs as well as Austrian 1823 with a "Dutch"-type handshape. Note that this is an inference using the logical framework of parsimony and should not be viewed as conclusive evidence that the "Austrian"-type replaced the "Dutch"-type. With respect to the age of the involved MAs and overall reconstructed history of SLs (see main text), it is equally probable that younger MAs of the Austrian-origin group (Danish 1808, Austrian 1823; see Figure S2) took over the more widespread "Dutch"-type replacing an ancestral "Austrian"-type typical for this lineage. Hence, there may be conflicts in the reconstructions shown in all three figures, each one using a different taxon subset and potentially a different subset of lingotypes. For example, in the mid-time network (Figure S3), we have an additional "Danish"-type and the "Dutch"-type is inferred as the original handshape for <*k*> in all MAs including this concept (hence, labelled as "O"). On the other hand, the "Russian"-type handshape used in contemporary Norwegian SL can be *deduced* to represent a borrowing or convergent development since older Norwegian MAs showed the "Danish"-type typical for the Danish subgroup within the Austrian-origin group ("Da. → Ru."; Figures S3 and S4).

4.2.1 Explanation of annotations and abbreviations used for lingotypes/handshapes in character maps

Inferred and deduced handshape mutations are annotated in the following character maps and relate to the lingotypes as labelled and defined in Supplementary Material, File "lists.xlsx". We define the abbreviations used in annotations below.

Annotation	Definition
B	Handshape that can be traced back to Bonet 1620, different from the handshape in Yebra's MA.
Y	Handshape that can be traced back to Yebra 1593, different from the handshape in Bonet's MA.

O	Original handshape: the handshape inferred as the original form of all covered European lineages. When SLs of different groups share a handshape with Bonet *and* Yebra, this handshape is labelled as "O". Per definition, "B", "Y" and "O" are mutually exclusive.
C	Cosmopolitan: the label indicates that this handshape is not only shared among different groups covered in the networks, but also in the British-origin and/or Afghan-Jordanian groups, for which our data set includes no historical MAs (hence, not part of any graph in Fig. 4 in the main paper, Figs S2–S4 in the appendix).
D	Derived handshape: a handshape shared by SLs of different lineages. Per definition, the label "D" is mutually exclusive with all other labels and was used when none of the other labels applied.
unique	Unique lingotype: handshapes restricted to a single SL.
†	Highlights that this particular handshape is only found in historical MAs.
Two letters	Handshapes (mostly) restricted to, exclusively found in a single main group.
At.	Austrian, Austrian-origin group
Da.	Danish, Danish subgroup within the Austrian-origin group
Du.	Dutch: used for handshapes of the French-origin group not shared by French SLs but found in the historical Dutch MAs (especially Dutch 1790, the oldest MA of the French-origin group in our data set).
Fr.	French, French-origin group
Ge.	German: used for handshapes of the Austrian-origin group not diagnostic for the Danish subgroup, not found in the oldest Austrian MAs, but found in historical German MAs
Po.	Polish, Polish group
Ru.	Russian, Russian group
Sp.	Spanish, Spanish-origin group: this can be a handshape exclusively found in Bonet, Yebra, and Spanish MAs, as well as a handshape found only in younger Spanish MAs but not Bonet or Yebra.
Sw.	Swedish, Swedish group

Table S4: Labels used in character mapping figures.

Coloured mutations refer to the accordingly coloured edge-bundle (taxon split) in the respective graphs here and in the main text: mutations in light grey (and smaller) font indicate unique mutations restricted to the respective OTU ("leaf") of the network; dark grey indicates the (inferred) handshape set (lingotypes) of a hypothetical common "ancestor".

4.2.2 Comprehensive character map for sign languages up to 1840

In the following figure, showing the character map for SLs up to 1840, the lists of concepts (bottom-left) highlight the handshapes shared (labelled subsequently as original/"O", or Spanish/"Sp.") and differing between the two oldest MAs (labelled as "B" or "Y") in our data set (Yebra 1593, Bonet 1620). The handshapes for the concept <*d*> are unique in both the Yebra and Bonet MAs; all other MAs show different handshapes (most show a handshape characteristic of the oldest MAs of the Austrian- and French-origin groups). Five concepts show no or singular variation.

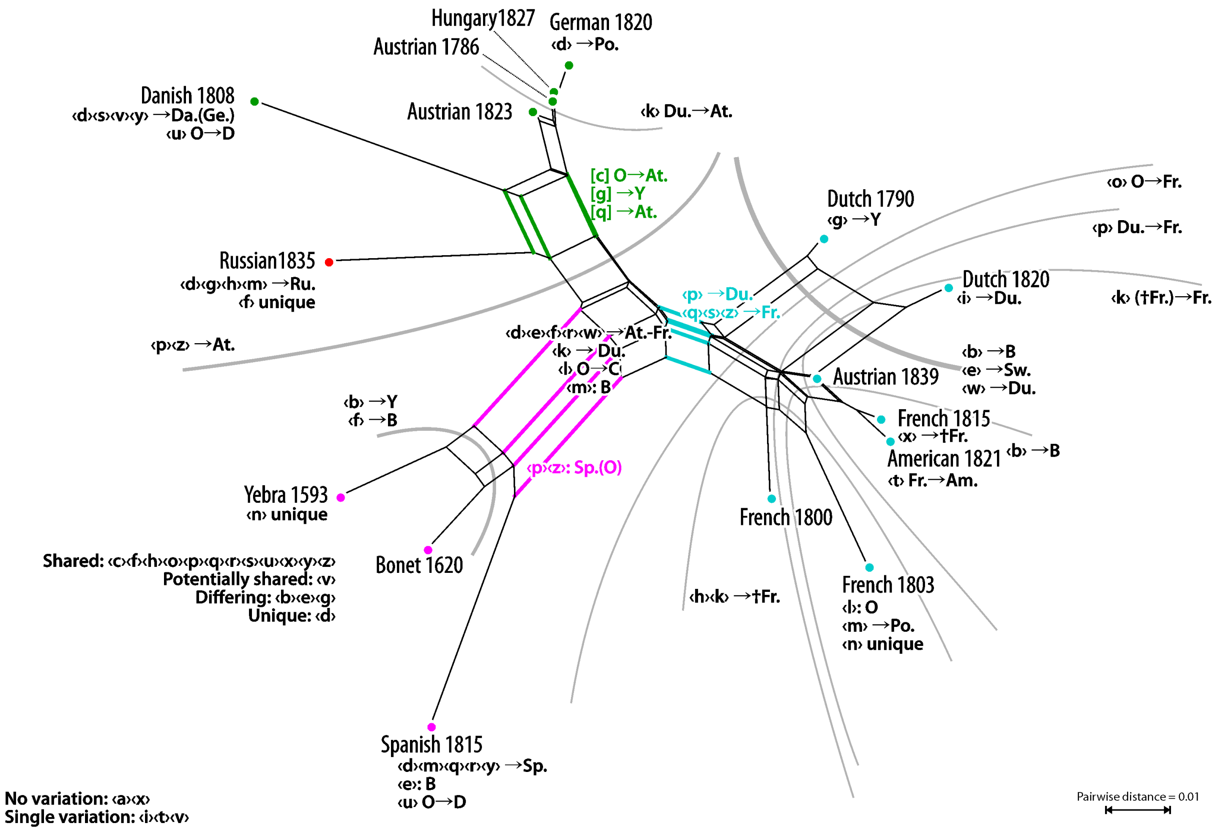

Figure S2: Character map for sign languages up to 1840.

4.2.3 Character map for sign languages from early 19th to mid-/late 20th century

Similar to the character map above, each lineage in the map shown in Figure S3 below can be characterised by group-specific handshapes (in coloured font). Note the striking difference between the diversification patterns in the French- vs. Austrian-origin group. The inflated fan-like structure of the NNet for the Austrian cluster relates to a gradual accumulation of lineage-specific handshapes and their subsequent modification (and/or) replacement in subgroups. Potential older sources (Spanish 1815, Russian 1835, German 1820, Danish 1808, French 1815, and American 1821 MAs) are included to link this taxon set with the one shown in Figure S2.

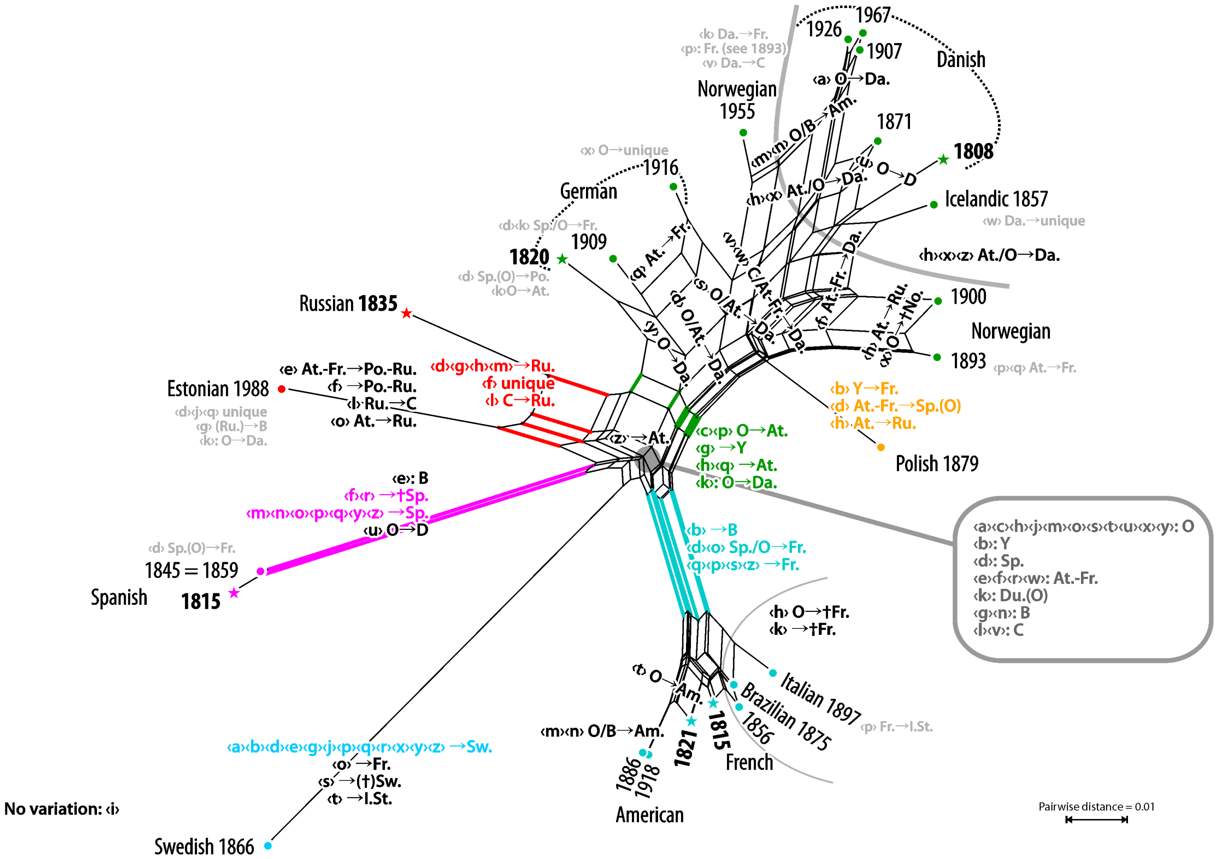

Figure S3: Character map for sign languages from early 19th to mid-/late 20th century.

4.2.4 Character map for contemporary sign languages

The taxon set of the character map for contemporary sign languages below also includes historical but post-war MAs (second half of the 20th century). In addition to lineage-specific handshapes, we find concepts directly linking the Polish and Austrian-origin group (coloured font) by shared handshapes. Note the high level of inferred single-SL (tip) mutations, especially in (phylogenetically) isolated SLs such as contemporary Norwegian (deduced mutations take into account also the historical Norwegian MAs, Figure S3), or Albanian SL.

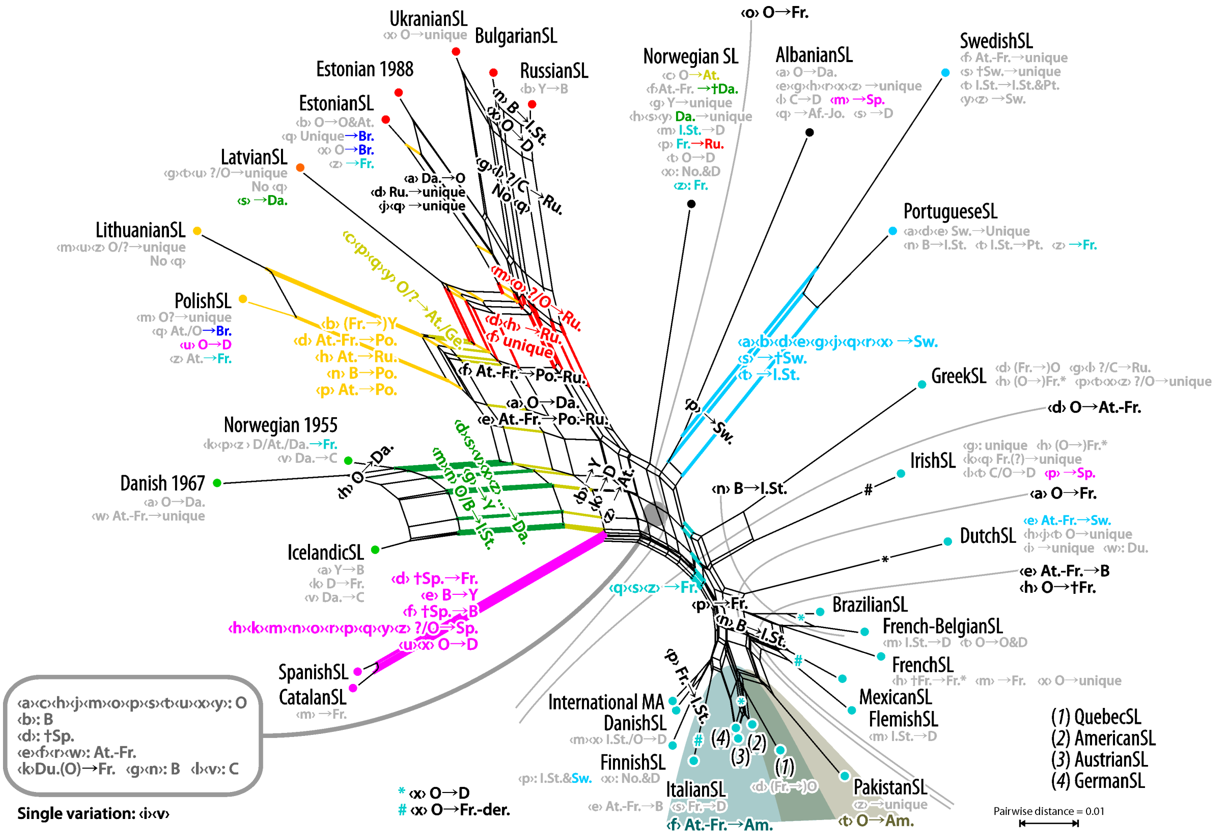

Figure S4: Character map for contemporary sign languages.

4.3 Split support

The following information about (alternative) split support has been extracted from the colour-annotated bootstrap CNets included as Splits-NEXUS-formatted files in the Supplementary Material (Folder "networks"; see also information provided in "lists.xlsx"). Split (edge/branch) support has been established under all three optimality criteria for phylogenetic trees using non-parametric BS: Maximum likelihood (ML), either corrected (ASC) or uncorrected (UNC) for ascertainment bias, least-squares via neighbour-joining (NJ), and maximum parsimony (MP).

The table below shows non-parametric BS support for competing relationships in the Polish and Russian groups with respect to Latvian SL. Figure 5 shows BS support for selected, partly overlapping neighbourhoods.

		ML-BS		NJ-BS	MP-BS
**Alternative type**	**Group**	ASC	UNC		
Terminal Unchallenged	Contemp. Cyrillic: Bulgarian SL, Russian SL, Ukranian SL	84	85	71	49
First-level					
CNet & NNet	Russian gr. incl. Russian 1835	37	37	27	54
CNet	Contemp. Russian gr. + Latvian SL	35	37	18	<15
CNet & NNet	Contemp. Russian gr. + Estonian SL	<15	<15	46	21
Second-level					
Preferred	Russian gr. incl. Russian 1835, excl. Latvian SL	18	17	40	39
CNet	Russian gr. incl. Latvian SL excl. Russian 1835	18	<15	30	<15
Deep split					
NNet-alternative	Latvian SL part of Russian gr.	41	43	33	39
NNet-alternative	Latvian SL part of Polish gr.	<15	<15	37	<15

Table S5: Summary on split support.

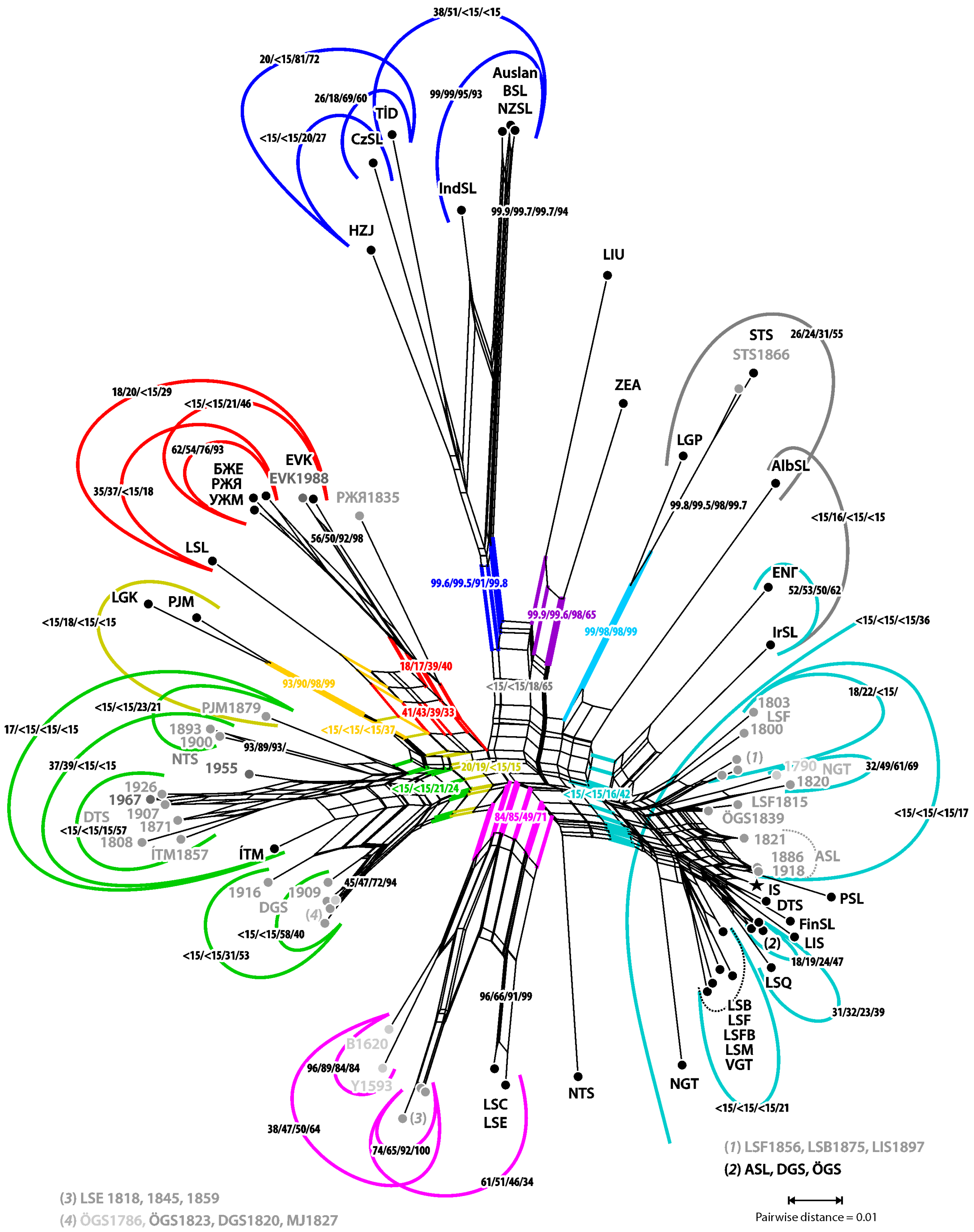

Figure S5: Split support for selected neighbourhoods/taxon-groups.

In Figure S5, a duplicate of Figure 3 in the main text, sign language names are abbreviated (see Tables S1 and S2); and colouring refers to the main SL groups (clockwise starting from top): blue – British-origin, violet – Afghan-Jordanian, light blue – Swedish, turquoise – French-origin, purple – Spanish, green – Austrian-origin, dark yellow – Polish, red – Russian. Olive refers to the Austrian-origin Polish neighbourhood. The numbers give the BS support of the corresponding split under ML, corrected and not corrected for ascertainment bias, MP and under LS via NJ.

The following four figures, Figure S6 A–D, show colour-annotated BS CNets using a cut-off of 20, i.e. only splits are shown that occur in at least 20% of the 10,000 BS pseudoreplicate trees inferred under maximum likelihood, maximum parsimony and with neighbour-joining (under the least-squares criterion). The edge-lengths in the CNets are proportional to the frequency of the corresponding split in the BS sample; trivial splits are collapsed.

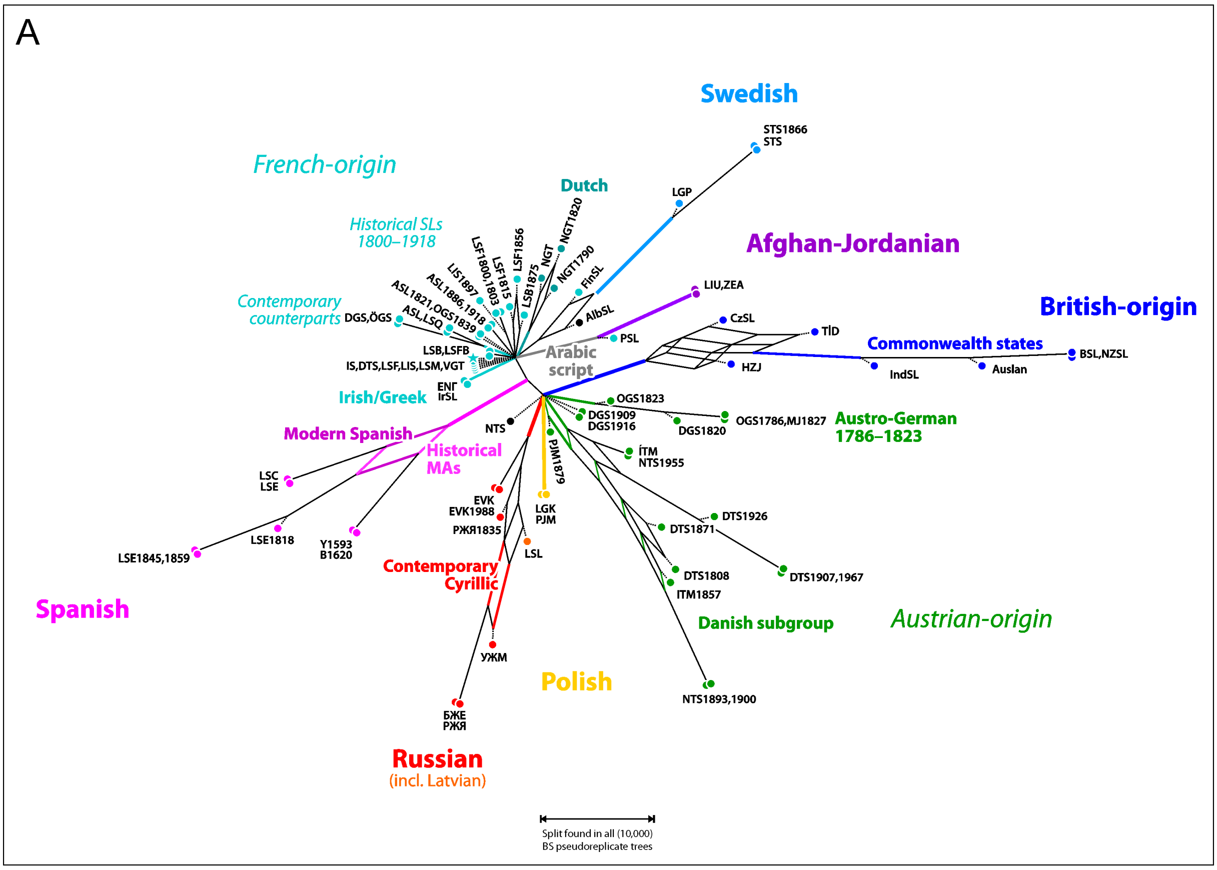

Figure S6A: ML-BS CNet, ascertainment bias corrected analysis.

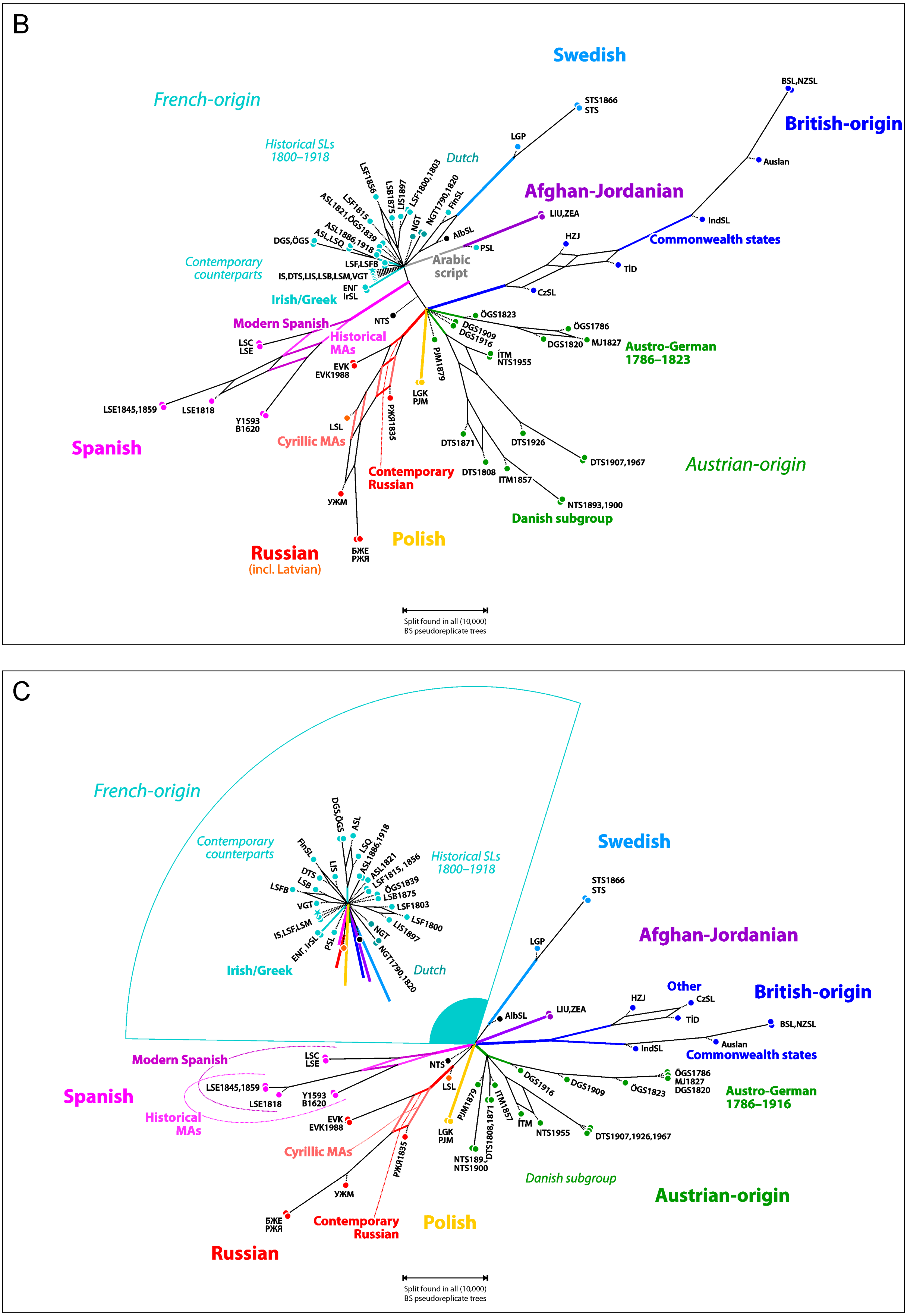

Figures S6B–C: B, ML-BS CNet, not corrected for ascertainment bias; C, MP-BS CNet.

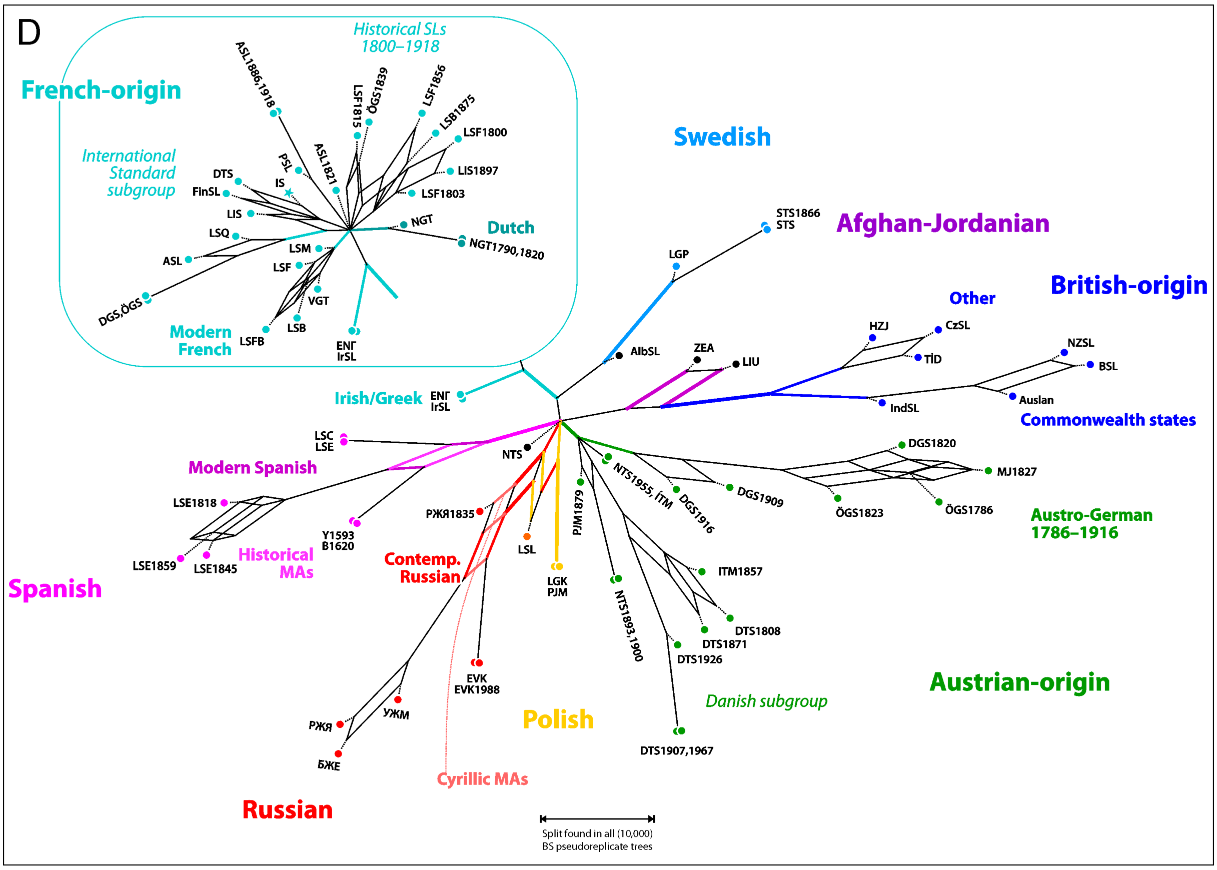

Figure S6D: NJ-BS CNet.

Recurring groups (i.e., subtrees comprising recurrent sets of OTUs in the BS pseudoreplicate tree samples) are annotated in **bold font**, when a corresponding split is represented in the CNet, and in *italics* when not resolved. The maximum dimension of box-like portions in the graphs is three; higher-dimensional boxes may only be found when using a lower cut-off value. This indicates that no MA in our data set is a complete mosaic and shows a strong signal for multiple (more than 2/3) sources. An exception may be Norwegian SL, the phylogenetic position of which remains unresolved under any criterion. Overall low support lacking alternatives hints towards resolution issues: few concepts define the relationship and inform the placement in the pseudoreplicate trees. Boxes seen in the CNets may reflect mixed affinities or potential ancestral-descendant relationships: modification within an SL group over time. For instance, under all optimality criteria, a prominent 2-dimensional box is found within the Spanish group, one split groups the historical MAs including the oldest MAs in our data set and likely sources for the Spanish group. A competing split, labelled “modern Spanish”, joins the MAs of the 19th century with their contemporary counterparts, Catalan and Spanish SL. The third possible alternative split, grouping the oldest with the youngest MAs is not realised. Such a situation is expected for gradual evolutionary pathways (in this case the Bonetian/Yebran MAs appear to have been modified into 19th Spanish MAs, which informed the contemporary SL MAs). Differences in split support for annotated groups (see also Supplementary Material, File “lists.xlsx” in Folder “analysis”, for a full list of competing and consecutive splits) relate to basic properties of our data matrix, which will need to be explored in more detail.

4.4 Evolution of early manual alphabet forms

The figure below compares MA forms from Yebra 1593 and Bonet 1620 with the earliest MAs in our sample from the Spanish (1815), French-origin (1800), and Austrian-origin (1786) lineages. Letters with circle backgrounds represent forms shared between Yebra and Bonet; for the five forms that differ between

Yebra and Bonet, square backgrounds represent Yebran forms and pentagons represent Bonetian forms. The Spanish, French-origin, and Austrian-origin areas of the figure track (i) forms that were coded as similar to the early sources, using pink circles, squares, and pentagons; (ii) forms that were coded as different compared with Yebra and Bonet, using dotted borders; (iii) forms that may have been derived, using a broken-line border and colour-coding that reflects which lineages shared the potential innovation; and, (iv) using a broken-line border and grey colour-coding, forms that may have been convergent or borrowed because they are shared widely across unrelated lineages.

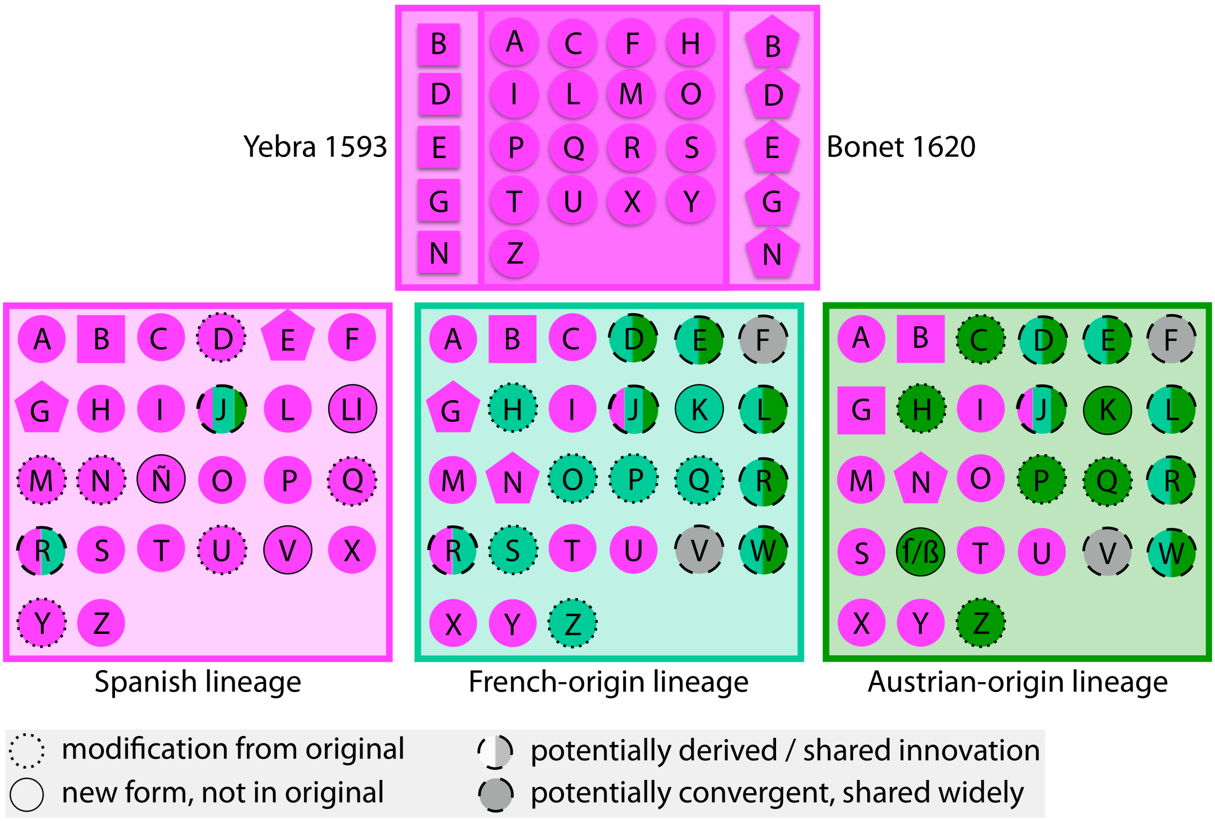

Figure S7: Comparing earliest MA forms for similarity.

References

Abramov, Igor A. (1993). "History of the deaf in Russia". In: *Looking back: A reader on the history of Deaf communities and their sign languages*. Ed. by Renate Fischer and Harlan Lane. Vol. 20. Hamburg: Signum-Verlag, 199–205.

Afghan National Association of the Deaf (2001). *Afghan Sign Language*. Kabul, Afghanistan: Comprehensive Disabled Afghans Program (UN).

Akerly, Samuel (1823). *Elementary exercises for the deaf and dumb*. New York: E. Conrad.

Albanian National Association of the Deaf, ed. (2019). *Gjuha e Shenjave Shqipe*. Online; accessed 28-March-2019. URL: `https://www.gjshsh.al/`.

Alle, Johann L. (1821). *Anleitung taubstumme Kinder in Schreiben, Lesen, Rechnen zu unterrichten und sie moralisch-gut und bürgerlich-brauchbar zu bilden*. Gmünd: Verlag d. K. Taubstummen-Lehranstalt.

Avdelningen för teckenspråk, Institutionen för lingvistik, ed. (2014). *Svenskt teckenspråkslexikon*. Online; accessed 28-March-2019. URL: `https://teckensprakslexikon.su.se/`.

Ballesteros, Juan Manuel and Francisco Fernández Villabrille (1845). *Curso elemental de instruccion de sordo-mudos. Parte segunda: Práctica de la enseñanza*. Madrid: Imprenta del Collegio de Sordo-mudos y Ciegos.

Bandelt, Hans-Jürgen, Vincent Macaulay, and Martin Richards (2000). "Median Networks: Speedy construction and greedy reduction, one simulation, and two case studies from human mtDNA". *Molecular Phylogenetics and Evolution* 16, 8–28.

Bergman, Brita and Elisabeth Engberg-Pedersen (2010). "Transmission of sign languages in the Nordic countries". In: *Sign languages*. Ed. by Diane Brentari. Cambridge language surveys. Cambridge: Cambridge University Press. Chap. 1.4, 74–94.

Bickford, J. Albert (2005). *The signed languages of Eastern Europe*. Tech. rep. SIL International and University of North Dakota.

Blanco, Ángel Herrero (2009). *Gramática didáctica de la Lengua de Signos Española (LSE)*. Spanish. Akademisk Forlag. Madrid: Ediciones SM.

Bonet, Juan Pablo (1620). *Reduction de las letras y arte para enseñar a ablar los mudos*. Madrid: Francisco Abarca de Angulo.

Boyes Braem, Penny and Christian Rathmann (2010). "Transmission of sign languages in Northern Europe". In: *Sign languages*. Ed. by Diane Brentari. New York: Cambridge University Press, 19–45.

Brien, David, ed. (1992). *Dictionary of British Sign Language: English*. London: Faber & Faber.

Bruun, Hans Rener (1900). *Det Norske Haandalphabet*. Oslo Museum, Byhistorisk samling. Online; accessed 3-April-2019. URL: https://digitaltmuseum.no/021017827436/det-norske-haandalphabet.

Carderera, Mariano (1859). *Apuntes sobre la educación elemental del sordomudo*. Madrid: Imprenta de Ramón Campuzano.

Castberg, Peter Atke (1818). *Forelæsninger over Døvstumme-Undervisningens Methode*. Danish. Copenhagen: L. Beetens Forlag.

Corazza, Serena and Luigi LeRose (2008). "Vergleich von Klassifikatoren in der Österreichischen und der Triestiner Gebärdensprache". In: *Gebärdensprachlinguistik und Gebärdensprachkommunikation. Referate der VERBAL-Sektion "Gebärdensprachlinguistik und -kommunikation" innerhalb der 34. Österreichischen Linguistiktagung*. Universität Klagenfurt, 31–36.

Czech, Franz Herrmann (1839). *Nothwendigkeit der allgemein einzuführenden Elementar-Bildung der Taubstummen, aus den Verhältnissen derselben zum Staate und zur Kirche hergeleitet und dargestellt*. 2nd ed. Vienna: P. P. Mechitaristen.

da Gama, Flausino José (1875). *Iconographia dos signaes dos surdos-mudos*. Rio da Janeiro: Typographia Universal de E. & H. Laemmert.

De Clerck, Goedele (2009). "The Flemish Deaf community and the challenges of breaking through barriers in the educational system". In: *Deaf people around the world: Educational and social perspectives*. Ed. by Donald F. Moores and Margery S. Miller. Washington, D.C.: Gallaudet University Press, 157–177.

de Ladébat, André-Daniel Laffon (1815). *Recueil des définitions et réponses les plus remarquables de Massieu et Clerc, sourds-muets, aux diverses questions quileur ontété faites dans les séances publiques de M. l'Abbé Sicard, à Londres: Auquel on a joint l'alphabet manuel des sourds-muets, le discours d'ouverture de M. l'Abbé Sicard, et une lettre explicative de sa méthode*. Online; accessed 28-March-2019. Cox et Baylis. URL: https://blogs.ucl.ac.uk/library-rnid/2013/07/04/sign-alphabet-exhibition-a-collection-of-the-most-remarkable-definitions-and-answers-of-massieu-and-clerc/.

de Yebra, Melchor (1593). *Libro llamado Refugium Infirmorum: Muy util y prouechoso para todo genero de gente : En el qual se contienen muchos auisos espirituales para socorro de los afligidos enfermos, y para ayudar à bien morir a los que estan en lo ultimo de su vida ; con vn Alfabeto de S. Buenauentura para hablar por la mano*. Madrid: Luys Sa[n]chez.

Døvstumme-Raadet (1926). *Ordbog i de døvstummes Tegnsprog*. Copenhagen: Herm. Rolsteds Bogtr.

Eberhard, David M., Gary F. Simons, and Charles D. Fennig, eds. (2019). *Ethnologue: Languages of the World*. 22nd. Dallas, Texas: SIL International.

Eesti Keele Instituut, ed. (2014). *Eesti Viipekeele –eesti keele sõnastiku*. Online; accessed 28-March-2019. URL: http://www.eki.ee/dict/viipekeel/.

Eriksson, Per (1998). *The history of deaf people: A source book*. Örebro, Sweden: Daufr.

Filippová, Eva and Andrea Hudáková (2016). "Czech Sign Language in contemporary Czech society". *International Journal of the Sociology of Language* 238, 85–103.

Fischer, Jutta, Janne Boye Niemelä, Kim Kanstrup Kjeldsen, and Thomas Troelsgård, eds. (2019). *Ordbog over Dansk Tegnsprog*. Online; accessed 28-March-2019. URL: http://www.tegnsprog.dk/.

Fleri, Viktor I. (1835). *Глухонемые, рассматриваемые в отношении к их состоянию и к способам образования, самым свойственным их при*. St. Petersburg: Типография А. Плюшара.

Fondation des Sourds du Québec, ed. (2019). *Dictionairre*. Online; accessed 28-March-2019. URL: http://www.courslsq.net/ewac/lsq/dictionary.php.

Fornari, Pasquale (1897). *Il sordomuto e la sua istruzione: Manuale per gli allievi e le allieve delle R. scuole normali, maestri, genitori e filantropi*. Milan: Ulrico Hoepli.

Geraci, Carlo (2015). "Italian Sign Language". In: *Sign languages of the world: A comparative handbook*. Ed. by Julie Bakken Jepsen, Goedele De Clerck, Sam Lutalo-Kiingi, and William B McGregor. Berlin, Germany and Preston, UK: Walter de Gruyter, Inc. and Ishara Press. Chap. III,I,19, 473–510.

Gordon, Joseph C. (1886). *The American manual alphabet*. Washington, D.C.: Bretano Bros.

Greftegreff, Irene, Tone-Britt Handberg, and Odd-Inge Schröder (2015). "Norwegian Sign Language". In: *Sign languages of the world: A comparative handbook*. Ed. by Julie Bakken Jepsen, Goedele De Clerck, Sam Lutalo-Kiingi, and William B McGregor. Berlin, Germany and Preston, UK: Walter de Gruyter, Inc. and Ishara Press. Chap. III,I,27, 649–676.

Hatzopoulou, Marianna (2008). "Acquisition of reference to self and others in Greek Sign Language: From pointing gesture to pronominal pointing signs". PhD thesis. Institutionen för lingvistik.

Hendriks, Bernadet (2008). *Jordanian Sign Language: Aspects of grammar from a cross-linguistic perspective*. Utrecht, The Netherlands: Landelijke Onderzoekschool Taalwetenschap.

Hendriks, Bernadet and Ulrike Zeshan (2009). "Sign Languages". In: *Encyclopedia of Arabic Language and Linguistics*. Ed. by Kees Versteegh. Vol. 4. Leiden: E.J. Brill, 222–235.

Hollaka, Józefa and Teofila Jagodzińskiego (2011). *Słownik mimiczny dla głuchoniemych i osób z nimi styczność mających*. Warszawa: Instytucie Głuchoniemych i Ociemniałych.

Hoyer, Karin (2007). "Albanian Sign Language: Language contact, International Sign, and gesture". In: *Sign languages in contact*. Ed. by David Quinto-Pozos. Vol. 13. Sociolinguistics in deaf communities. Washington, D.C.: Gallaudet University Press.

Hudáková, Andrea (2008). *Prstová abeceda pro tlumočníky*. Česká komora tlumočníků znakového jazyka.

Johnston, Trevor (2014). *Auslan signbank*. Online; accessed 27-March-2019. URL: http://www.auslan.org.au/spell/twohanded.html.

Jørgensen, Johannes (1907). *De døvstummes Haandalfabet og 280 af de almindeligste Tegn*. Copenhagen: Alfred Jacobsens grafiske Etablissement.

Kusters, Marieke (2017). "Intergenerational responsibility in deaf pedagogies". In: *Innovations in deaf studies: The role of deaf scholars*. Ed. by Annelies Kusters, Maartje De Muelder, and Dai O'Brien. New York: Oxford University Press. Chap. 10.

Kuurojen Liitto ry ja Kotimaisten kielten tutkimuskeskus, ed. (1998). *Suomalaisen viittomakielen perussanakirja*. Kotimaisten kielten tutkimuskeskuksen julkaisuja 104. Helsinki: KL Support Oy.

Lampropoulou, Venetta (1994). "The history of deaf education in Greece". In: *The deaf way: Perspectives from the international conference on deaf culture*. Ed. by Carol J. Erting, Robert C. Johnson, Dorothy L. Smith, and Bruce D. Snider. Washington, D.C.: Gallaudet University Press, 239–249.

Latvian Association of the Deaf, ed. (2016). *Alfabēts*. Online; accessed 28-March-2019. URL: http://zimjuvaloda.lv/lv/alphabet.

Lee, Soo Choo (1999). "A Project for Deaf People in Afghanistan". unpublished report.

Leeson, Lorraine, John I. Saeed, and Carmel Grehan (2015). “Irish Sign Language (ISL)”. In: *Sign languages of the world: A comparative handbook*. Ed. by Julie Bakken Jepsen, Goedele De Clerck, Sam Lutalo-Kiingi, and William B McGregor. Berlin, Germany and Preston, UK: Walter de Gruyter, Inc. and Ishara Press. Chap. III,I,18, 449–472.

LeMaster, Barbara (2002). “What difference does difference make? Negotiating gender and generation in Irish Sign Language”. In: *Gendered practices in language*. Ed. by Sarah Benor, Mary Rose, Sharma Devyani, Julie Sweetland, and Qing Zhang. Standford, CA: CSLI Publications.

Lietuvos kurčiųjų ir neprigirdinčiųjų ugdymo centras and Metodinių Priemonių Rengimo ir gestotyros skyrius, eds. (2012). *Lietuvių Gestų Kalbos žodynas*. Online; accessed 28-March-2019. URL: http://gestai.ndt.lt/pirstu-abecele.

List, Johann-Mattis (2017). “A web-based interactive tool for creating, inspecting, editing, and publishing etymological datasets”. In: *Proceedings of the 15th Conference of the European Chapter of the Association for Computational Linguistics. System Demonstrations*. Valencia: Association for Computational Linguistics, 9–12.

List, Johann-Mattis, Simon Greenhill, Tiago Tresoldi, and Robert Forkel (2018). *LingPy. A Python library for quantitative tasks in historical linguistics*. Jena: Max Planck Institute for the Science of Human History. URL: http://lingpy.org.

Long, Joseph Schuyler (1918). *The sign language: A manual of signs, illustrated*. 2nd ed. Des Moines, IA: Robert Henderson.

Lydell, Thomas, ed. (2018). *Spreadthesign*. URL: https://www.spreadthesign.com/.

Magarotto, Cesare (1996). *Vocabolario della Lingua Gestuale Italiana dei sordi*. Armando Editore.

Martí Mora, Francisco de Paula (1815). *Alfabeto manual para la instrucción de los sordo-mudos del Real Colegio de Madrid*. Biblioteca de Catalunya. Online; accessed 3-April-2019. Barcelona. URL: http://www.bnc.cat/Media/Images/Alfabeto-manual-para-la-instruccion-de-los-sordo-mudos-del-Real-Colegio-de-Madrid.

May, Joseph (1789). *Anleitung zum Unterrichte der Taubstummen nach der Lehrart des Herrn Abbe de l'Epée zu Paris, nebst einer Nachricht von dem kaiserl. königl. Taubstummeninstitute in Wien*. Vienna: k. k. Taubstummeninstitutsbuchdruckerey.

McCagg Jr., William O. (1993). “Some problems in the history of deaf Hungarians”. In: *Deaf history unveiled: Interpretations from the new scholarship*. Ed. by John Vickrey Van Cleve. Washington, D.C.: Gallaudet University Press. Chap. 15, 252–271.

McKee, David, Rachel McKee, S. Pivac Alexander, L. Pivac, and M. Vale (2011). *Online dictionary of New Zealand Sign Language*. Online; accessed 28-March-2019. Wellington. URL: https://www.nzsl.nz/.

Mörser, I.L. and H.D. Guyot (1790). *Het alphabet manuel of het vingerspraak voor doven en stommen*. Groningen.

Norske Døves Landsforbund (1955). *Håndalfabet for Døve*. Sverresborg Trøndelag Folkemuseum, Trøndelag Folkemus. Online; accessed 3-April-2019. Trøndelag. URL: https://digitaltmuseum.no/011022836818/bilde.

Nyegaard, Andreas C. (1871). *De Døvstummes Haandalphabet samt et Udvalg af deres lettere Tegn sammenstillet, tegnet, graveret og udgivet af en Forening af Dövstumme*. Danish. Copenhagen: Kongelige Dövstumme-Institut.

Parisot, Anne-Marie, Julie Rinfret, Suzanne Villeneuve, and Amélie Voghel (2015). “Quebec Sign Language”. In: *Sign languages of the world: A comparative handbook*. Ed. by Julie Bakken Jepsen, Goedele De Clerck, Sam Lutalo-Kiingi, and William B McGregor. Berlin, Germany and Preston, UK: Walter de Gruyter, Inc. and Ishara Press. Chap. III,I,30, 701–728.

Parkhurst, Stephen and Dianne Parkhurst (2003). *Lexical comparisons of signed languages and the effects of iconicity*. University of North Dakota Session 47. SIL International.

Paulsson, Per (1866). *Historik öfver folkunderwisningen i Sverige från äldsta till närvarande tid*. Stockhom: Adolf Bonnier.

Pelissier, Pierre (1856). *Iconographie des signes*. Paris: Paul Dupont.

Perelló, Jorge and Juan Frigola Masclans (1998). *Lenguaje de signos manuales*. Madrid: CIE Inversiones Editoriales Dossat.

Plann, Susan (1997). *A silent minority: Deaf education in Spain, 1550-1835*. Berkeley: University of California Press.

Plum, Ole Munk, Sofus Kjær, Asger Holm, and Orla A. Odde (1967). *Håndbog i tegnsprog*. Copenhagen: Danske Døves Landsforbund.

Quinto-Pozos, David (2008). “Sign language contact and interference: ASL and LSM”. *Language in Society* 37.2, 161–189.

Reuschert, E. (1909). *Die Gebärdensprache der Taubstummen und die Ausdrucksbewegungen der Vollsinnigen*. Leipzig: Kommissions-Verlag von H. Dude.

Riemann, G. (1916). *Taubstumm und blind zugleich: Pädagogische und psychologische Darbietungen*. 2nd ed. Berlin: Schriftenvertriebsanstalt GmbH.

Rubino, F., A. Hayhurst, and J. Guejlman (1975). *Gestuno: International Sign Language of the Deaf*. Carlisle: British Deaf Association.

Sabir Khan, Nabeel, Adnan Shahzada, Saleem Ata, Adnan Abid, Yaser Khan, Shoaib Farooq, Tahir Mushtaq, and Inayatullah Khan (2014). “A Vision Based Approach for Pakistan Sign Language alphabets Recognition”. *La Pensée* 76.03, 274–285.

Sapountzaki, Galini (2015). “Greek Sign Language”. In: *Sign languages of the world: A comparative handbook*. Ed. by Julie Bakken Jepsen, Goedele De Clerck, Sam Lutalo-Kiingi, and William B McGregor. Berlin, Germany and Preston, UK: Walter de Gruyter, Inc. and Ishara Press. Chap. III,I,11, 318–334.

Schalber, Katarina (2015). “Austrian Sign Language”. In: *Sign languages of the world: A comparative handbook*. Ed. by Julie Bakken Jepsen, Goedele De Clerck, Sam Lutalo-Kiingi, and William B McGregor. Berlin, Germany and Preston, UK: Walter de Gruyter, Inc. and Ishara Press. Chap. III,I,3, 105–128.

Schembri, Adam, Kearsy Cormier, Trevor Johnston, David McKee, Rachel McKee, and Bencie Woll (2010). “Sociolinguistic variation in British, Australian and New Zealand Sign Languages”. In: *Sign languages*. Ed. by Diane Brentari. New York: Cambridge University Press, 476–498.

Schwarzer, Anton (1827). *Lehrmethode zum Unterrichte der Taubstummen in der Tonsprache für Lehrer*. Buda: Universitäts-Schriften.

Sicard, Roch Ambroise Cucurron (1803). *Cours d'instruction d'un sourd-muet de naissance, pour servir à l'éducation des sourds-muets, et qui peut être utile à celle de ceux qui entendent et qui parlent*. 2nd ed. Paris: Le Clere.

Sigurðsson, Sigfús (1857). *Fingramálsstafróf*. Akureyri: Jósef Grímsson.

Sonnemans, Bruno (2016). *LSFB asbl: An online dictionary and newspaper in LSFB free access of videos*. Online; accessed 28-March-2019. URL: http://www.lsfb.be/, dicto.lsfb.be, flash5.lsfb.be.

Spencer, Matthew, E. A. Davidson, A. C. Barbrook, and C. J. Howe (2004). “Phylogenetics of artificial manuscripts.” *Journal of Theoretical Biology* 227, 503–511.

Statped, ed. (2016). *Norsk tegnordbok*. Online; accessed 28-March-2019. URL: https://www.minetegn.no/Tegnordbok-2016/.

Svendsen, Conrad (1893). *De Døvstumme, deres Opdragelse i Hjem og Skole*. Kristiania: H. Aschehoug & Co.s Forlag.

Tabak, John (2006). *Significant gestures: A history of American Sign Language*. Westport, Connecticut: Praeger Publishers.

Tennant, Richard A. and Marianne Gluszak Brown (1998). *The American Sign Language handshape dictionary*. Gallaudet University Press.

Thorvaldsdóttir, Kristín Lena and Valgerður Stefánsdóttire (2015). “Icelandic Sign Language”. In: *Sign languages of the world: A comparative handbook*. Ed. by Julie Bakken Jepsen, Goedele De Clerck, Sam Lutalo-Kiingi, and William B McGregor. Berlin, Germany and Preston, UK: Walter de Gruyter, Inc. and Ishara Press. Chap. III,I,16, 409–429.

Toom, Koostaja R (1988). *Kõnelevad käed: Eesti Viipekeele sõnastik*. Estonian. Tallinn: Tartu riiklik ülikool eesti NSV kurtide ühing.

unknown, Author (1799-1800). *Alphabet, manuel-figure des sourds-muets de naissance*. Online; accessed 28-March-2019. Paris. URL: https://blogs.ucl.ac.uk/library-rnid/2017/03/17/alphabet-manuel-figure-des-sourds-muets-de-naissance-an-viii-1799-1800/.

Šarac Kuhn, Ninoslava, Tamara Alibašić Ciciliani, and Ronnie B. Wilbur (2006). "Phonological parameters in Croatian Sign Language". *Sign Language & Linguistics* 9.1, 33–70.

Van Cleve, John V., ed. (1987). Vol. 3. McGraw-Hill.

van Heijningen Bosch, M. (1823). *Berigt, houdende eenige wenken over het eerste onderwijs aan doofstommen: Benevens het hand-alphabet, in het koper gegraveerd*. 2nd ed. Groningen.

Vasishta, Madan M. (2011). "Social situations and the education of deaf children in India". In: *Deaf around the world: The impact of language*. Ed. by Gaurav Mathur and Donna Jo Napoli. Oxford: Oxford University Press. Chap. 11, 352–358.

Venus, Michael (1823). *Das kais. kön. Taubstummen-Institut in Wien, dessen Entstehung, Erweiterung und gegenwärtiger Zustand*. Vienna: Anton Strauß.

Viktorivna, Culbida Svitlana (2010). "Use of sign language in the study of the non-student". Ukrainian. PhD thesis. Kiev, Ukraine: Institute of Special Pedagogy.

Vlaams GebarentaalCentrum vzw, ed. (2012). *Vertaalwoordenboek*. Online; accessed 28-March-2019. URL: http://gebaren.ugent.be/.

Von Der Lieth, Lars (1967). *Dansk døve-tegnsprog*. Akademisk Forlag.

Williams, Howard G. and Polina Fyodorova (1993). "The origins of the St. Petersburg institute for the deaf". In: *Looking back: A reader on the history of Deaf communities and their sign languages*. Ed. by Renate Fischer and Harlan Lane. Vol. 20. Hamburg: Signum-Verlag, 295–305.

Wojda, Piotr (2010). "Transmission of Polish sign systems". In: *Sign languages*. Ed. by Diane Brentari. New York: Cambridge University Press, 131–147.

Xavier, André Nogueira and Regiane Pinheiro Agrella (2015). "Brazilian Sign Language (Libras)". In: *Sign languages of the world: A comparative handbook*. Ed. by Julie Bakken Jepsen, Goedele De Clerck, Sam Lutalo-Kiingi, and William B McGregor. Berlin, Germany and Preston, UK: Walter de Gruyter, Inc. and Ishara Press. Chap. III,I,4, 129–158.

Zeshan, Ulrike (2003). "Aspects of Türk Isaret Dili (Turkish Sign Language)". *Sign Language & Linguistics* 6.1, 43–75.

Zwitserlood, I.E.P. (2010). "Laat je vingers spreken. NGT en vingerspelling". *Levende Talen Magazine* 97.2, 46–47.

Łacheta, Joanna, Małgorzata Czajkowska-Kisil, Jadwiga Linde-Usiekniewicz, and Paweł Rutkowski, eds. (2016). *Korpusowy słownik Polskiego Języka Migowego*. Online; accessed 28-March-2019. Warsaw: Faculty of Polish Studies, University of Warsaw. URL: http://www.slownikpjm.uw.edu.pl/en.
